# Patterns of attention deficit in relapsing and progressive phenotypes of multiple sclerosis

**DOI:** 10.1038/s41598-023-40327-x

**Published:** 2023-08-10

**Authors:** Farinaz Tabibian, Kiarash Azimzadeh, Vahid Shaygannejad, Fereshteh Ashtari, Iman Adibi, Mehdi Sanayei

**Affiliations:** 1 Neurosciences Research Center, Isfahan University of Medical Sciences, Isfahan, Iran; 2 Center for Translational Neuroscience, Isfahan University of Medical Sciences, Isfahan, Iran; 3 Department of Neurology, Isfahan University of Medical Sciences, Isfahan, Iran; 4 School of Cognitive Sciences, Institute for Research in Fundamental Sciences (IPM), Tehran, Iran

**Keywords:** Attention, Multiple sclerosis

## Abstract

Behavioral aspects and underlying pathology of attention deficit in multiple sclerosis (MS) remain unknown. This study aimed to clarify impairment of attention and its relationship with MS-related fatigue. Thirty-four relapse-remitting MS (RRMS), 35 secondary-progressive MS (SPMS) and 45 healthy controls (HC) were included. Results of psychophysics tasks (attention network test (ANT) and Posner spatial cueing test) and fatigue assessments (visual analogue scale and modified fatigue impact scale (MFIS)) were compared between groups. In ANT, attentional network effects were not different between MS phenotypes and HC. In Posner task, RRMS or SPMS patients did not benefit from valid cues unlike HC. RRMS and SPMS patients had less gain in exogenous trials with 62.5 ms cue-target interval time (CTIT) and endogenous trials with 250 ms CTIT, respectively. Total MFIS was the predictor of gain in 250 ms endogenous blocks and cognitive MFIS predicted orienting attentional effect. Executive attentional effect in RRMS patients with shorter disease duration and orienting attentional effect in longer diagnosed SPMS were correlated with MFIS scores. The pattern of attention deficit in MS differs between phenotypes. Exogenous attention is impaired in RRMS patients while SPMS patients have deficit in endogenous attention. Fatigue trait predicts impairment of endogenous and orienting attention in MS.

## Introduction

Multiple sclerosis (MS) is the primary cause of non-traumatic disability in young adults^1^. Two-third of MS patients suffer from cognitive impairment which reduces their quality of personal, social, and professional life^2^. Although there is increasing evidence for the importance of cognitive comorbidities in MS, consensus on appropriate objective assessment and treatment has not been reached so far^3^ and the course of these comorbidities can only be poorly predicted^4–6^. Attention, the process through which the brain selects information for further processing^7,8^, is one of the main affected cognitive domains in adults with MS^9^. Their common complaints are about switching attention and keeping up with a specific stimulus when there are distractors nearby^10^. Moreover, attention is also frequently impaired in pediatric MS patients^11–13^.

Various factors can affect attention of MS patients. Both neuropsychological and psychophysics studies have highlighted the role of disease phenotype in impairment of attention; Some studies have shown more frequent and severe difficulties in progressive forms of the disease, while others point to contrary findings^14–16^. However, patterns of attention deficit in different MS phenotypes have not been studied in details.

MS-related fatigue has been also suggested as a possible effective factor on attention^17,18^. Fatigue is the most common and debilitating symptom in MS patients^19^ and has been shown to correlate with changes in brain networks e.g., salience network, which are responsible for attentional processes^20^. Moreover, attention tests have been proposed as a valid measure of cognitive fatigue in MS^21,22^. However, the results regarding this relationship are inconsistent; As reviewed by Golan et al., Some studies have found a negative correlation between fatigue and attention, while in others no association was observed after considering cofounding variables^23^. Conflicting findings and unclear pathophysiology of these conditions in MS^24^ highlight the need for further evaluation.

Most studies have used neuropsychological tests to assess attention of MS patients^2,25,26^. These tests only give a general evaluation of attention without distinguishing between different forms of impairment, which can partly explain the inconsistency of previous results. On the other hand, psychophysics tasks enable a straightforward detailed assessment of attention with minimal influence from other cognitive domains. Also, different psychophysics tasks can engage different forms of attention, which can be used to categorize attentional impairment.

Gonzalez-Rosa et al., were the first group applying an attention psychophysics task—Posner spatial cueing test^27^—in MS patients. In addition to poorer task performance in the patient group, those with benign form of the disease showed higher attentional deterioration than relapse-remitting MS (RRMS)^16,28^. Later on, Urbanek et al., conducted attention network test (ANT) in MS patients^29^. They found a significant lower *alerting* effect in RRMS patients compared to healthy control (HC) and no differences in *orienting* and *executive* effects^30^. In ANT, each network carries out a function similar to its name; *Alerting* network achieves and maintains alert state, *orienting* network selects information from sensory input, and *executive* network resolves conflict among responses. Although stability and reliability of ANT results were shown across time in RRMS patients, attentional network deficits were not similarly reproduced^31–34^. Roth et al., recruited secondary progressive MS (SPMS) as well as RRMS and applied ANT. They analyzed the data from different perspectives and found that the attentional deficit was confined to *alerting* network and only in SPMS patients^35^. Among psychophysics studies of attention in MS, only a few have evaluated different disease phenotypes and no study has investigated the role of fatigue.

In this study, we applied 2 psychophysics tasks i.e., ANT and Posner spatial cueing test, in order to provide a clearer picture of attentional impairment in RRMS and SPMS phenotypes of MS. As far as we know, this is also the first study to investigate the relationship between MS-related fatigue and attention in more details by measuring separately the trait and the state of fatigue and evaluating attention using psychophysics tasks.

## Materials and methods

### Participants

Sixty-nine MS patients (34 with RRMS and 35 with SPMS phenotype) of MS day clinic at Kashani Hospital, Isfahan, Iran, who had following criteria, were recruited in a full-census manner: had been diagnosed based on McDonald criteria 2017^36^; were between 18 to 55 years old; had been diagnosed longer than 5 and shorter than 15 years; had no history of acute clinical relapse or treatment with corticosteroids in the last 2 months; had normal or corrected-to-normal vision; performed 9-hole peg test (9-HPT) in less than 45 s for each hand^37^; did not have any history of brain surgeries, major neurologic and psychiatric disorders (stroke, epilepsy, brain tumor, central nervous system infection, major depressive disorder, bipolar disorder, schizophrenia, and substance abuse), chronic systemic disorders (diabetes, renal failure, liver failure, and chronic obstructive pulmonary disease), possible causes of fatigue (anemia, hypothyroidism, hyperthyroidism, vitamin-D deficiency, and sleep disorders), and taking medications that possibly affect cognition (antiepileptics, antidepressants, antipsychotics, and antihistamines). An increased impairment in one functional system score to 3 or in total EDSS score to 1.5 was considered as significant disability. Confirmed progression in SPMS patients was defined as sustained significant disability for at least 6 months. Results of 9-HPT of dominant hand based on seconds were adjusted for age and gender according to the recently published norms for further analysis^38^. Forty-five HC who were demographically comparable to the patient group and had no first-degree relatives diagnosed with MS were included as well.

### Procedure

Subjects sat in front of a 15″ cathode ray tube monitor at ~ 48 cm distance to perform psychophysics tasks, after the instructions were given to them written and verbally. Persian version of modified fatigue impact scale (MFIS) and Montreal cognitive assessment (MoCA) test were also taken from subjects. MFIS questionnaire has 21 items, concerning the frequency of fatigue experienced in the past 4 weeks (trait of fatigue), and gives a *total* score and three subscores of *physical*, *cognitive*, and *psychosocial*^39^. MoCA test is a 30-point cognitive screening tool evaluating attention, memory, visuospatial abilities, executive function, language, and orientation that gives a *total* score^40^.

### Psychophysics tasks

ANT and Posner spatial cueing test^29,41^ were designed in MATLAB version R2016a (MathWorks Inc., Natick, Massachusetts, USA) using PsychToolbox extension-3^42,43^. Before and after each of the six blocks of the tasks, a 10 cm line marked by numbers from 0 to 100 in steps of 25, visual analogue scale (VAS)^44^, was shown to the participant to select a score for the level of fatigue experienced at that moment (state of fatigue) by clicking on the desired number. Mean VAS score was computed as the average of all reported VAS scores through each task. Out of all subjects, 34 patients (16 RRMS, 18 SPMS) and 12 HC participated in both psychophysics tasks.

#### ANT

**Figure 1:**
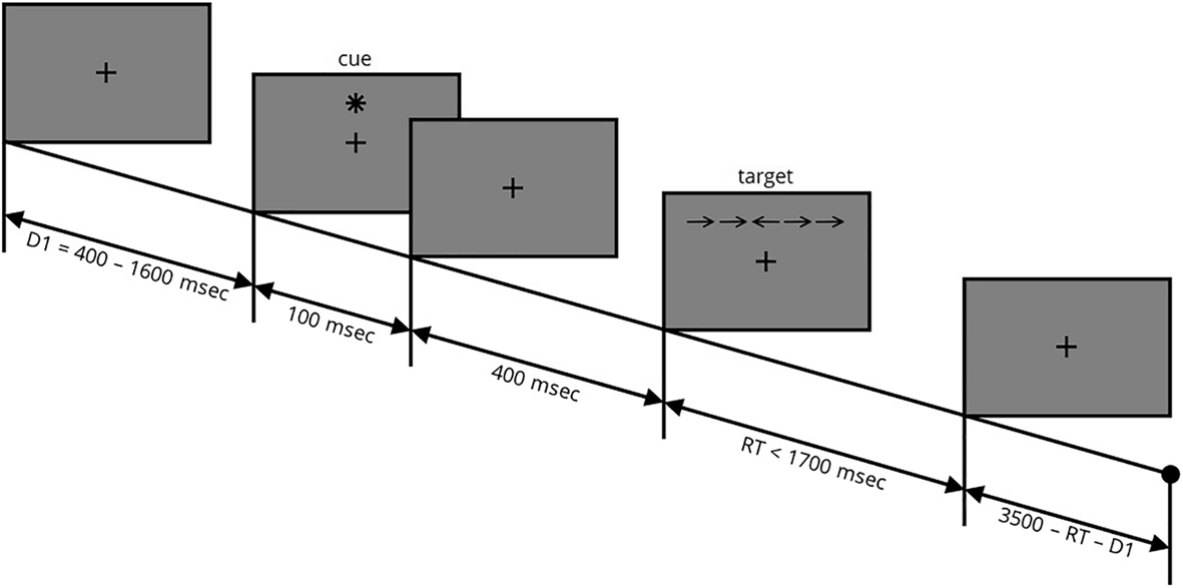
Attention network test. The fixation cross (size = 2.3°) was shown on the screen for a random time between 400 and 1600 ms. Then, the cue (no cue, center cue, double cue, or spatial cue) appeared on the screen for 100 ms. After 400 ms blank period, the target was presented (neutral, congruent, or incongruent). Subjects had 1700 ms to determine the orientation of central arrow by pressing right arrow or left arrow key on the keyboard. The inter-trial interval was calculated based on the first random delay (D1) and the reaction time (RT). In the example here, the presented cue is *spatial* and the target is *incongruent*. *ms* millisecond.

Each block consisted of 48 randomly-presented trials (4 cue types × 6 target types × 2 target positions). Subjects were asked to keep fixation throughout the trials. One of four types of cues was presented on each trial (Fig. 1). *No cue* was the continuation of displaying the fixation cross for assessing the state of non-alertness, whereas *central cue*, was an asterisk with similar size and position with fixation cross to alert the subject. *Double cue* were two asterisks, 1° above and below the fixation cross, to partially orient the subject besides alerting them. S*patial cue* was an asterisk 1° above or below the fixation cross that completely assessed the state of alerting and spatial orienting of the participant. After a 400 ms delay, one of six following types of 3° targets was presented at the position of spatial cue. Target was consisted of a right or left directed arrow at the center and two lines or arrows on the right and left sides of the central one (line or arrow length: 0.55°, separation distance: 0.06°). The first (and second) *neutral* target had 4 flanker lines and a right (and left) directed arrow at the center. The third and fourth type had 5 similar rightward or leftward arrows called *congruent* targets. The last two *incongruent* ones had 4 flanker arrows with the opposite direction relative to the central arrow. The subject had to press the right or left arrow key on the keyboard to report the direction of central arrow as soon as possible.

#### Posner spatial cueing test

**Figure 2:**
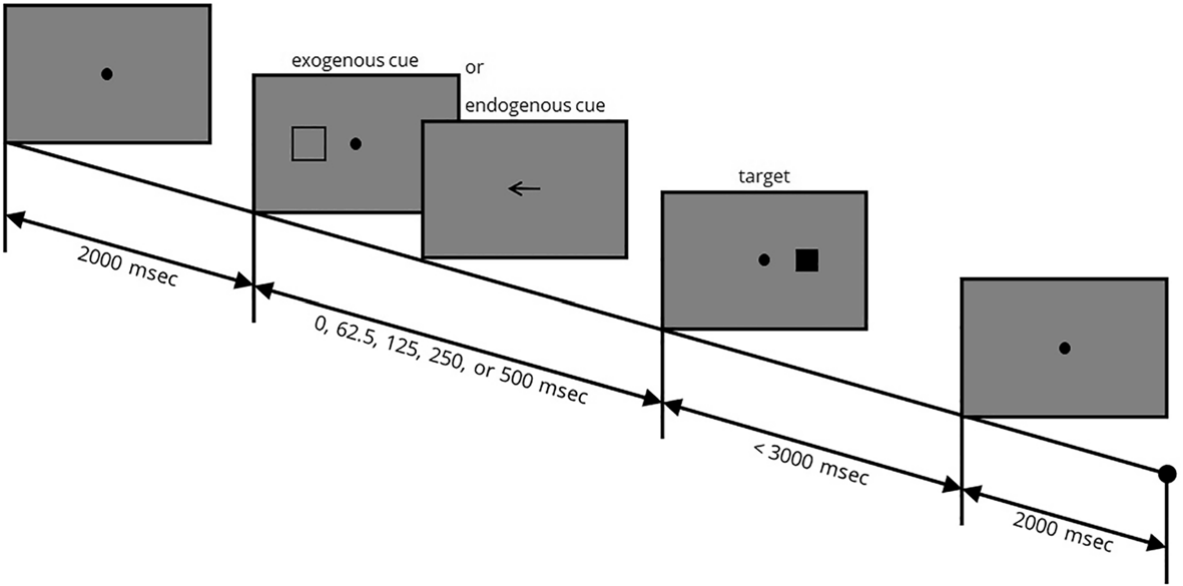
Posner spatial cueing test. The fixation point (radius = 0.4°) was presented on the screen for 2000 ms. Then, an exogenous or endogenous cue was presented to subject for a pseudorandom cue-target interval time. A black solid square (size = 7°) appeared next as the target. The subject had 3000 ms time for reporting the location of target by pressing the right arrow or left arrow key on the keyboard. Inter-trial time was 2000 ms. This example shows an *invalid* trial with two possible examples of *endogenous* and *exogenous* cue. *ms* millisecond.

Each trial of this task began with presenting a fixation point and subjects were asked to keep fixation throughout the task (Fig. 2). Target was a solid black square presented at 7° right or left of the fixation point. Subject had to press the right or left arrow key on the keyboard as soon as the target was seen. In the first three blocks to assess *exogenous* attention, cue was an empty black square (size = 10°) presented at the same location as the target in 80% of non-neutrally cued trials (*valid* trials), or on the opposite site of the fixation point compared to the following target in the remaining 20% of the trials (*invalid* trials). In the last 3 blocks, the cue was a right or left directed arrow (size = 10°) at the center of screen to evaluate *endogenous* attention. A plus sign (size = 10°) presented at the position of fixation point acted as a *neutral* cue in both types of exogenous and endogenous tasks, where the probability of target presentation on the right or left side was 50%. Out of 70 trials in each block, 25 (and 25) trials had right (and left) directed arrow or right-sided (left-sided) empty square, and the remaining 20 trials had plus sign as the neutral cue. Cue-target interval times (CTIT, 0, 62.5, 125, 250, or 500 ms) were presented equally in each block in a random order.

In both tasks, the time from target presentation to key press by subject was defined as reaction time (RT) which was only reported for correct trials. Error rate (ER) was defined as the percent of wrong responses to the sum of wrong and correct responses.

### Statistical analysis

Statistical analysis was performed using MATLAB version R2021b. Whenever data did not follow the normal distribution (tested by Anderson–Darling test), Kruskal–Wallis test with Tukey Kramer post hoc and Spearman correlation were used for comparing groups and evaluating associations, respectively. Comparing proportions were done by applying χ^2^ test of independence. Hierarchical regression was performed using generalized linear model with gamma or normal distribution and canonical link to determine effects, interactions, and predictive variables. Moreover, area under receiver operating curve (ROC) was measured for evaluation of discriminatory capacity. Significance level was 0.05 in all tests.

### Ethics approval

This study was performed in line with the principles of the Declaration of Helsinki. Approval was granted by the Ethics Committee of Isfahan University of Medical Sciences, Isfahan, Iran (Ethics committee code: IR.MUI.MED.REC.1400.229).

### Informed consent

Informed consent was obtained from all included subjects prior to their participation in the study.

## Results

### ANT

**Figure 3:**
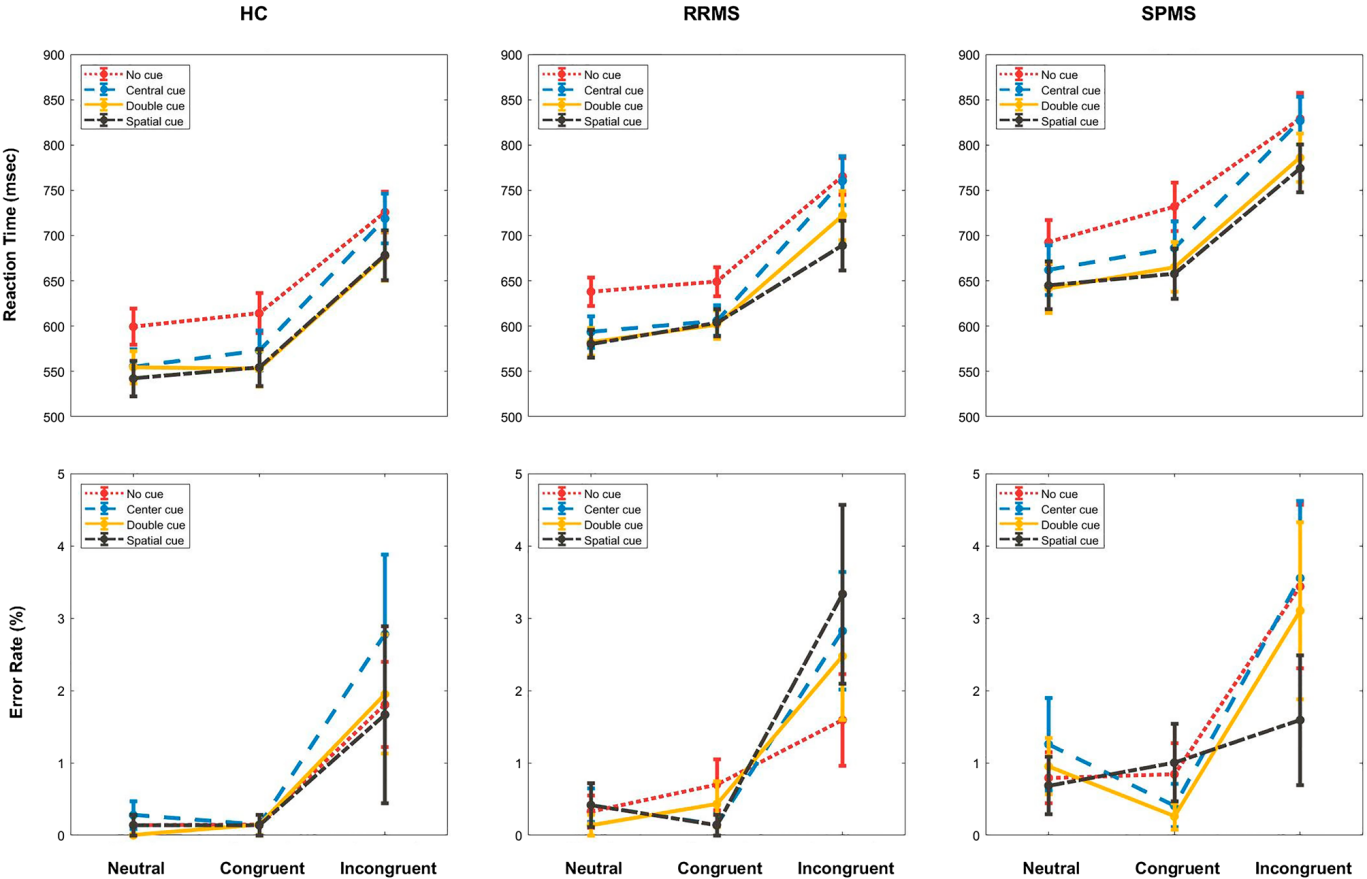
Reaction time and error rate of participants in attention network test. Reaction time (top) and error rate (bottom) are presented in millisecond and percent, respectively, for HC (left), RRMS (middle) and SPMS (right) groups. Error bars are between subject ± 1 standard error of mean. Results in each group (HC, RRMS, and SPMS) are separated by the type of cue (no cue, center cue, double cue, and spatial cue) and target (neutral, congruent, and incongruent). In almost all trial types, reaction time of SPMS patients was significantly longer than HC, and error rate was similar between groups. *HC* healthy control, *RRMS* relapse-remitting multiple sclerosis, *SPMS* secondary progressive multiple sclerosis, *ms* millisecond.

Demographic and clinical characteristics of participants of this task are summarized in Table 1 in addition to disease modifying drugs (DMD) of patients which are found in Supplementary Table S1. RT and ER of participants are shown for each group in Fig. 3. Total RT was significantly longer in SPMS patients compared to HC (χ^2^ = 9.3, p < 0.007). Unlike ER, almost all cue-target specific RTs were also significantly longer in SPMS group versus HC (Supplementary Table S2). By assessing the effect of group on RT or ER separately and with consideration of clinical characteristics, group showed significant effect on RT (t = − 3.4, p < 0.002). Also, RT of incongruent trials were longer than neutral trials (t = − 4.5, p < 0.001), and ER was higher in incongruent compared to neutral trials (t = 2.6, p < 0.009) in all groups. In HC age had significant effect on RT (t = − 6.6, p < 0.001); however, different cues, disease duration, EDSS, or 9-HPT had no effect on either RT or ER in all groups (p > 0.06).

**Table 1:** Demographic and clinical characteristics of participants in attention network test.

Demographic or clinical characteristic	HC	RRMS	SPMS	p-value*
Number	30	30	32	NA
Age (years)	37.2 ± 11.4	34.4 ± 7.8	38.4 ± 5.9	p = 0.195
Gender	16:14	20:10	25:7	p = 0.119
Education (years)	13.3 ± 4.7	12.9 ± 4.6	11.9 ± 3.8	p = 0.172
Disease duration (years)	NA	8.0 ± 2.8	10.3 ± 3.3	(RRMS vs. SPMS) p = 0.002*
EDSS Score	NA	1.5 ± 1	3.5 ± 2.5	(RRMS vs. SPMS) p = 0.006*
Total MFIS Score	20.1 ± 14.9	33.1 ± 18.5	31.9 ± 17.8	(RRMS vs. HC) p = 0.018*
Physical MFIS Score	8.4 ± 6.7	15.9 ± 8.9	17.8 ± 9.2	(RRMS and SPMS vs. HC) p = 0.001*
Cognitive MFIS Score	9.6 ± 6.6	14.2 ± 8.6	10.5 ± 7.5	p = 0.092
Psychosocial MFIS Score	2.1 ± 2.1	2.8 ± 2.2	3.6 ± 2.4	p = 0.0656
Mean VAS Score	38.9 ± 27.5	34.0 ± 29.1	37.2 ± 27.9	p = 0.7219
Total MoCA Score	27.5 ± 2.0	26.7 ± 1.7	24.7 ± 3.7	(SPMS vs. HC) p = 0.0012*

All data are presented as mean ± standard deviation, except gender that is reported as female:male and EDSS score that is reported as median ± interquartile range. Significant p-values (p-value < 0.05) of comparing groups (HC, RRMS, SPMS) using Kruskal–Wallis test are marked by * and groups with significant differences following Tukey Kramer post hoc analysis are noted in parenthesis.

*HC* healthy control, *RRMS* relapse-remitting multiple sclerosis, *SPMS* secondary progressive multiple sclerosis, *EDSS* expanded disability status scale, *NA* not applicable, *MFIS* modified fatigue impact scale, *VAS* visual analogue scale, *MoCA* Montreal Cognitive Assessment.

**Figure 4:**
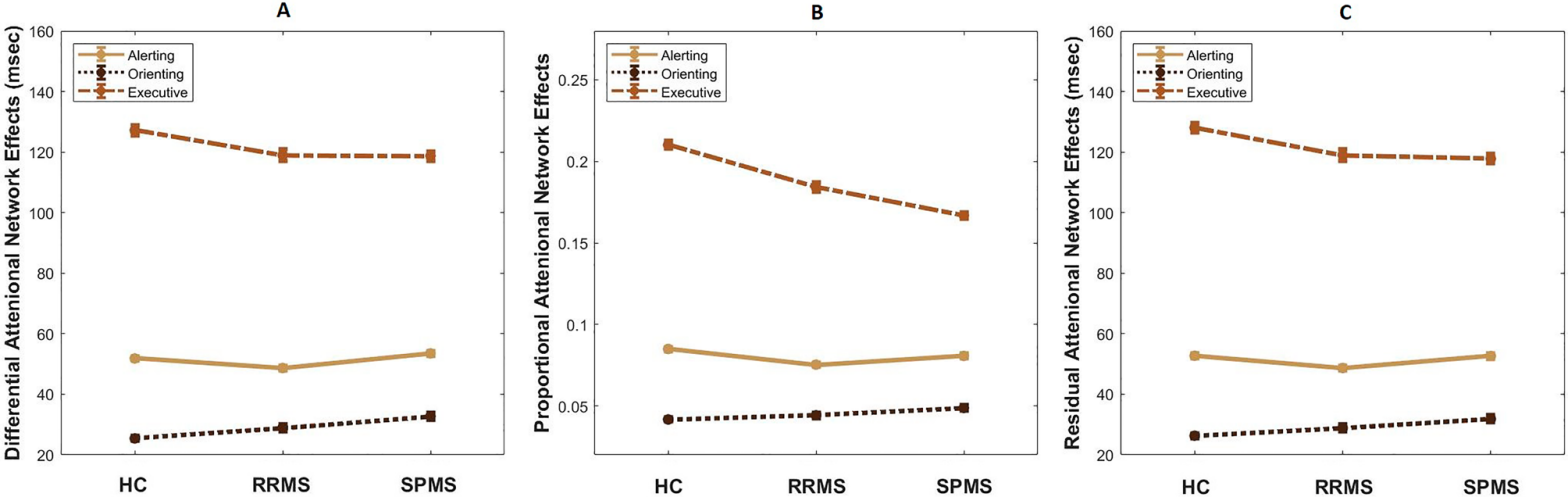
Attentional network effects of participants in attention network test. Differential attentional effects (**A**) for HC, RRMS and SPMS were calculated as follows: alerting effect = mean RT of no cue trials − mean RT of double cue trials; orienting effect = mean RT of center cue trials − mean RT of spatial cue trials; executive effect = mean RT of incongruent trials − mean RT of congruent trials. When differential effects are divided by mean RT of all trials or adjusted through linear regression by mean RT of no cue-neutral trials, proportional (**B**) and residual (**C**) effects were obtained, respectively. There is no difference between groups (HC, RRMS, and SPMS) in all attentional network effects (alerting, orienting, and executive), regardless of the measurement method (differential, proportional, residual). Error bars are between subject ± 1 standard error of mean. *HC* healthy control, *RRMS* relapse-remitting multiple sclerosis, *SPMS* secondary progressive multiple sclerosis, *ms* millisecond.

Calculation of attentional network effects was based on previous studies^29–31^. Differential alerting effect is the subtraction of mean RT of trials with double cue from trials with no cue, while differential orienting effect is the difference between mean RT of trials with spatial cue and center cue. Differential executive effect is measured by subtracting the mean RT of congruent trials from incongruent ones (Fig. 4A). Proportional effects (Fig. 4B) are differential effects divided by mean RT of all trials, and residual effects (Fig. 4C) are differential effects adjusted by mean RT of trials with no cue and neutral targets using linear regression model. Differential, proportional, and residual attentional network effects, including *alerting*, *orienting*, and *executive*, were not statistically different between groups (p > 0.1) (Supplementary Table S3).

**Figure 5:**
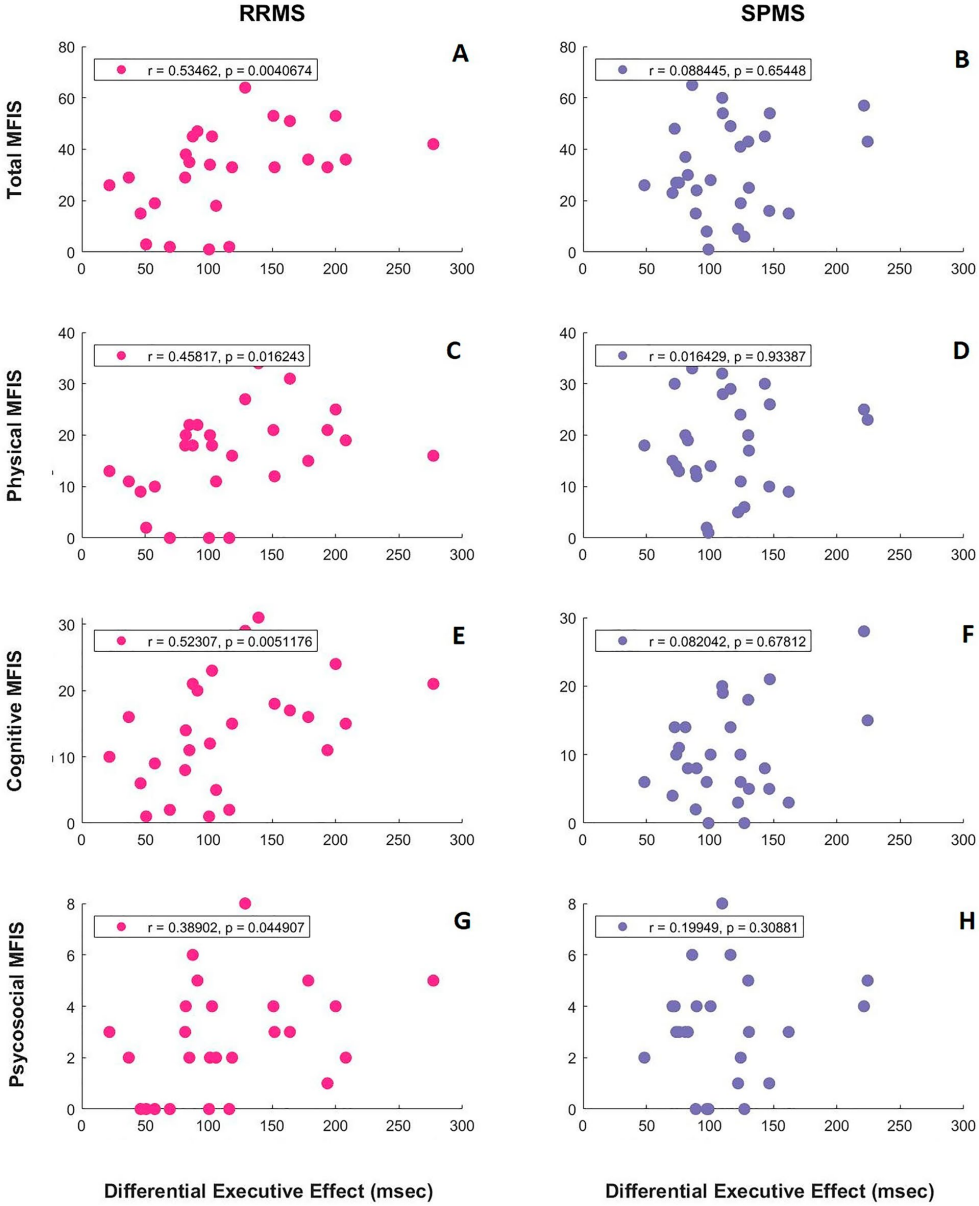
Relationship between differential executive attentional effect and modified fatigue impact scale scores in attention network test. Each point in each plot represents differential executive effect and modified fatigue impact scale (MFIS) score (top row) and its subscores (the rest) from one patient (left: RRMS, right: SPMS). Reported r and p were calculated by Spearman correlation with significance level of 0.05. Positive correlation was observed in RRMS patients. *RRMS* relapse-remitting multiple sclerosis, *SPMS* secondary progressive multiple sclerosis, *ms* millisecond.

By evaluating the association of MFIS scores and attentional network effects, differential executive effect was found to be positively correlated with MFIS scores in RRMS (not SPMS) patients (Fig. 5). Proportional or residual executive effects also showed the same trend (r = 0.3, p < 0.05). Moreover, when RRMS and SPMS patients were divided to 2 groups separately based on the median of disease duration, EDSS, or 9-HPT in each phenotype, shorter diagnosed RRMS patients showed the previously mentioned positive correlation between executive attentional effect and MFIS scores (r = 0.6, p < 0.03). Also, a negative correlation was found in longer diagnosed SPMS patients between orienting attentional effect and MFIS scores (r = − 0.6, p < 0.04). By EDSS analysis, a significant positive correlation was observed in RRMS patients with more severe disability between executive attention and total, physical, and cognitive MFIS scores (r = 0.6, p < 0.05). Same trend was also shown by 9-HPT analysis in RRMS patients with weaker hand function between executive attention and total and physical MFIS scores (r = 0.6, p < 0.05).

To assess predictors of attentional network effects, we considered group (RRMS and SPMS), MFIS (total and cognitive, separately) or VAS scores, disease duration, and 9-HPT or EDSS score consecutively in hierarchical regression analyses. At the level of adding disease duration to predictors of differential executive effect (group and cognitive MFIS score), group showed significant main effect (t = 2.2, p < 0.04) and significant interaction with disease duration (t = − 2, p < 0.05). For differential orienting effect, at the level of adding cognitive MFIS score to group, the main effect of cognitive MFIS (t = 2.8, p < 0.008) and its interaction with group (t = − 2.5, p < 0.02) were statistically significant. Regression analyses using total MFIS, VAS, or 9-HPT revealed no significant predictive effect.

### Posner spatial cueing test

Demographic and clinical characteristics of participants of this task are summarized in Table 2 in addition to DMDs of patients which are found in Supplementary Table S1. RT and ER of participants are shown for both exogenous and endogenous tasks in Supplementary Figs. S1 and S2. Only ER at 125 ms (χ^2^ = 9.1, p < 0.01) and 500 ms (χ^2^ = 6.6, p < 0.04) CTIT were significantly higher in SPMS versus RRMS patients in the exogenous task. Classifying trials by type of the cue or CTIT, showed that trials with 500 ms CTIT were significantly shorter than trials with 0 ms CTIT in all groups (p < 0.02). In addition, valid trials were significantly shorter than invalid trials in both endogenous (χ^2^ = 9.9, p < 0.002) and exogenous (χ^2^ = 6.3, p < 0.02) tasks in HC, while this was not observed in RRMS or SPMS patients (p > 0.07).

**Table 2:** Demographic and clinical characteristics of participants in Posner spatial cueing test.

Demographic or clinical characteristic	HC	RRMS	SPMS	p-value *
Number	27	20	21	NA
Age (years)	41.4 ± 10.1	37.2 ± 6.3	39.8 ± 5.8	p = 0.213
Gender	17:10	14:6	17:4	p = 0.397
Education (years)	12 ± 4.6	12.1 ± 5.1	12 ± 4.7	p = 0.999
Disease duration (year)	NA	8.3 ± 2.9	11.9 ± 3.8	(SPMS vs. RRMS) p = 0.001*
EDSS Score	NA	2 ± 1	4 ± 2	(SPMS vs. RRMS) p = 0.001*
Total MFIS Score	20.3 ± 16.3	35.8 ± 23.8	37.1 ± 19.9	(SPMS vs. HC) p = 0.032*
Physical MFIS Score	9.9 ± 7.7	17.8 ± 11.2	20.1 ± 9.3	(SPMS vs. HC) p = 0.013*
Cognitive MFIS Score	8.6 ± 7.4	14.9 ± 10.8	13.1 ± 9.1	p = 0.128
Psychosocial MFIS Score	1.7 ± 2.3	3.1 ± 2.7	3.8 ± 2.5	(SPMS vs. HC) p = 0.044*
Mean VAS Score	38.3 ± 25.6	42.4 ± 27.4	39.2 ± 22.3	p = 0.931
Total MoCA Score	27 ± 2.3	26.4 ± 2.1	24.1 ± 4.0	(SPMS vs. HC) p = 0.024*

All data are presented as mean ± standard deviation, except gender that is reported as female:male and EDSS score that is reported as median ± interquartile range. Significant p-values (p-value < 0.05) of comparing groups (HC, RRMS, SPMS) using Kruskal–Wallis test are marked by * and groups with significant differences following Tukey Kramer post hoc analysis are noted in parenthesis.

*HC* healthy control, *RRMS* relapse-remitting multiple sclerosis, *SPMS* secondary progressive multiple sclerosis, *EDSS* expanded disability status scale, *NA* not applicable, *MFIS* modified fatigue impact scale, *VAS* visual analogue scale, *MoCA* Montreal Cognitive Assessment.

**Figure 6:**
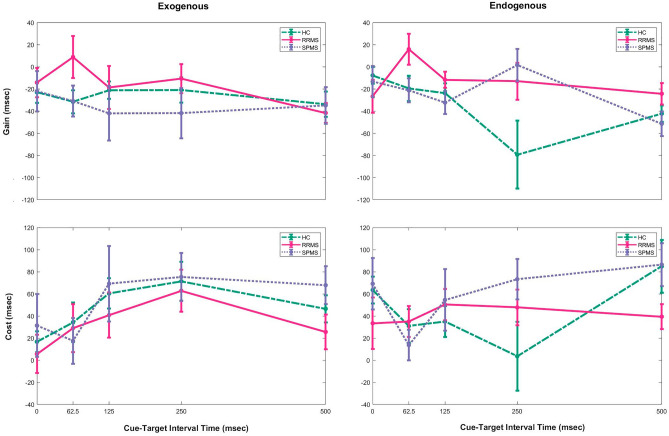
Gain and cost of participants in Posner spatial cueing test. Gain was defined as the difference between mean reaction time of valid trials and neutral trials. Cost was measured by subtracting the mean reaction time of neutral trials from invalid trials. Both were presented in milliseconds. Error bars are between subject ± 1 standard error of mean. Results are classified by the type of block (exogenous on left and endogenous on right) and cue-target interval time (0, 62.5, 125, 250, and 500 ms). Compared to HC, less significant gain was obtained by RRMS patients in 62.5 ms CTIT in the exogenous task, and by SPMS patients in 250 ms CTIT in the endogenous task. *HC* healthy control, *RRMS* relapse-remitting multiple sclerosis, *SPMS* secondary progressive multiple sclerosis, *ms* milliseconds.

We defined *gain* as the difference between mean RT of valid trials and neutral trials (smaller the number, higher the gain), and *cost* as the mean RT of invalid trials subtracted by neutral trials (smaller the number, lower the cost). Gain and cost, averaged over all trials of endogenous or exogenous tasks, were statistically similar between groups (p > 0.1). However, taking CTIT into account (Fig. 6), revealed that RRMS patients had less gain in 62.5 ms CTIT in the exogenous task (χ^2^ = 6, p < 0.05) and SPMS patients had less gain in 250 ms in the endogenous task (χ^2^ = 6.9, p < 0.04), compared to HC (Supplementary Table S4). By plotting ROC curve and calculating area under curve (AUC) for gain in 62.5 ms CTIT in exogenous task (0.6738) and 250 ms CTIT in endogenous task (0.6015), acceptable ability of these two measures to discriminate RRMS from SPMS phenotype was shown (Supplementary Fig. S3).

Evaluating the association between gain of 250 ms in the endogenous task and clinical characteristics, significant correlation was observed in SPMS patients with EDSS (r = 0.5, p < 0.04) and 9-HPT (r = − 0.5, p < 0.05). We did not find any significant relationship for gain of 62.5 ms in the exogenous task.

Hierarchical regression analyses were applied to determine predictive variables for endogenous (at 250 ms CTIT) or exogenous (at 62.5 ms CTIT) gain. Group (RRMS and SPMS), MFIS (total and cognitive, separately) or VAS scores, disease duration, and 9-HPT or EDSS score were considered in this order. When the dependent variable was gain at 250 ms in the endogenous task, and predictors were group, total MFIS score, disease duration, and EDSS, total MFIS showed significant main effect (t = − 2.2, p < 0.04) as well as an interaction with EDSS (t = 2.1, p < 0.05). Replacing total MFIS with VAS or cognitive MFIS, EDSS with 9-HPT, or using gain at 62.5 ms in exogenous task as the dependent variable did not lead to any significant effect.

## Discussion

In ANT, SPMS patients reacted to each type of cues and targets slower than HC albeit with comparable number of correct responses and differences between trials because of the presence of distractors. This was not the case for RRMS patients, which raises the question of whether poorer performance of SPMS could be due to more severe disability or longer disease duration versus RRMS. However, disability—measured by EDSS and 9-HPT—and disease duration had no effect on RT or ER. This observation was in line with previous studies, in which more severe cognitive impairment was still remarkable in MS patients with progressive forms of disease after considering their disability status and duration of disease^14^.

Regarding attention of MS patients, some previous psychophysics studies also have reported longer RT than HC that might not accompany higher ER^30,33,34^, tough only Roth et al., have enrolled SPMS phenotype^35^. They observed general slowing as well as more false responses in SPMS compared to HC. In addition, they were the only group who applied all three methods of measuring attentional networks (*differential*, *proportional*, and *residual*) and found out *alerting* impairment in SPMS patients. According to our results, no matter which method for measurement was used, RRMS and SPMS phenotypes did not differ from HC in their ability to alert, orient, and execute in presence of distractors in environment. This difference could be explained by the larger and more homogenous sample population in our study which did not include patients at both ends of the spectrum of disease duration and in turn disability.

In Posner spatial cuing test, although performance regarding RT or ER was not poorer in MS patients, neither RRMS nor SPMS benefited from valid cues in contrast to HC. Gonzalez et al., the only previous group that studied attention in MS with Posner paradigm, found that benign forms of disease did not show validity effect (RT of valid trials subtracted by RT of invalid trials^41^) probably due to a divided attention condition instead of an oriented spatial attention^16^. We extended their finding to a broader spectrum of MS including both RRMS and SPMS phenotypes. Also, we divided validity effect into *gain* and *cost* mathematically and did not find any difference in gain or cost between groups, which was at odd with what was observed about validity effect. To investigate this matter further, we looked at cost and gain at different CTITs.

For the first time to best of our knowledge, in this study, endogenous and exogenous spatial attention were studied separately in each patient with MS. In case of a voluntary goal, endogenous attention, and in the presence of an unexpected external stimulus in environment, exogenous attention is deployed^45^. Previous experiments have shown that exogenous attention is deployed in windows shorter than 100 ms, while endogenous attention come into use around 300 ms^46^. In our study, 62.5 ms and 250 ms CTIT in Posner spatial cuing test represent almost the peak effects of exogenous and endogenous attention, respectively.

The pattern of attention deficit differs from RRMS to SPMS phenotype. Comparing gain and cost between groups by considering CTIT, and the AUC for gain in 62.5 ms in exogenous and 250 ms in endogenous task, revealed that Posner spatial cueing test can differentiate RRMS from SPMS phenotype. Exogenous attention is impaired in RRMS patients, while SPMS patients have deficit in endogenous attention. That might be one of the reasons why we did not observe validity effect in neither of MS phenotypes. Current evidences suggest that endogenous and exogenous spatial attention have distinct neural basis besides different behavioral effects^47,48^, e.g., dorsal fronto-parietal regions and frontal to parietal direction of connectivity modulation are engaged in endogenous attention, while more ventral fronto-parietal network and preceding parietal activity have been observed in exogenous attention^49^. Along the course of MS from early to late stages, brain pathologies vary from focal white matter to more cortical and diffuse demyelinating lesions^50^. Thus, distinct neural substrates of endogenous and exogenous attention might link to distinguishable brain pathologies of RRMS and SPMS, waiting for further studies for its evaluation. As reviewed by Brochet et al., few previous studies have compared cognitive impairment between RRMS and SPMS patients using neuropsychological tests^14^, which have mostly reported more frequent and more severe attentional impairment in SPMS compared to RRMS. However, the patterns or mechanisms underlying cognitive differences between RRMS and SPMS phenotypes have not been studied in detail.

Our results also support the idea that two aspects of disease progression in MS (physical and cognitive) could interact with each other^51^; As SPMS patients with more severe disability had more impaired endogenous attention. This was shown not only by the EDSS score but also by the 9-HPT, which might be a better indicator of disability in psychophysics studies that are dependent on hand function.

The relationship between trait of fatigue and cognition in MS has been previously studied^17,52–55^. Findings regarding this relationship are inconsistent, such that only some studies suggest an association between the level of fatigue and impairment in cognitive domains including attention. Moreover, it has been shown that the interplay between fatigue and cognition is not completely independent of other clinical features such as comorbidities^23^.

In this study in RRMS patients, cognitive fatigue could predict better performance in orienting attention, whereas in SPMS, particularly longer diagnosed patients, cognitive fatigue was negatively correlated with orienting attention. This result can be partly explained by the dual regulation system of fatigue in MS patients^56^. According to Ishii et al., fatigue can activate an inhibitory response during challenging tasks. In the meantime, an increased brain activation compensates for inhibitory effects of fatigue to ensure cognitive performance, as seen in RRMS patients. However, when brain damages are too great, as in progressive forms of disease, this compensatory response may be insufficient and the inhibitory effect of fatigue prevails^57^.

As another result, cognitive fatigue was negatively correlated with the ability to maintain attention in presence of distractors and conflicting information only in RRMS patients. This could be justified by previous studies which have suggested executive failure as the main characteristic of cognitive fatigue^58,59^. The reason why this was not seen in SPMS patients, could be the different pathophysiological factors that are associated with fatigue in different phenotypes of MS, suggested in previous studies^60,61^.

How fatigue affects attention has not been widely studied. Few clues have been obtained through neuroimaging studies which assessed attention and fatigue simultaneously. Thalamic subregions, hippocampus, and supramarginal area have been shown to be in relation with both fatigue and attention in MS patients^62–64^. Also, similar brain changes underlying attention deficit and MS-related fatigue have been revealed separately, such as structural changes in cortico-striato-thalamo-cortical loops, functional alterations in prefrontal and parietal regions, and dopamine dysregulation^65–68^. Based on possible shared brain mechanisms and behavioral association of fatigue and attentional impairment, Hanken et al., proposed attention as the signature of MS-related fatigue^22^. Recent recommended cognitive phenotypes of MS proposed by de Meo et al., is consistent with this idea as only patients with *severe executive/attention* phenotype had high level of fatigue compared to others^69^. Specific details and pathways through which fatigue and attentional deficits in MS interact with each other requires large effort by conducting more mechanistic studies, since the pathophysiology of either of them is not clearly known yet.

Cross-sectional nature of this study limited causal interpretations and assessment of attention, fatigue, and their relationship through time. Moreover, we excluded MS patients with different comorbidities and those who experienced recent clinical relapse. Also, DMDs of patients and their objective state of other cognitive functions (e.g., information processing speed through symbol digit modalities test, executive function through paced auditory serial additional test) or psychiatric health (e.g., depression screening through questionnaires) were not considered in the statistical analysis. Longitudinal studies recruiting MS patients of different phenotypes in all stages of disease that enroll in psychophysics tasks of different cognitive domains beside neuroimaging evaluation are warranted, to fill the gaps and answer remaining questions.

### Supplementary Information

Supplementary Information.

## Data Availability

The datasets used and/or analyzed during the current study are available from the corresponding author on reasonable request*.*
