# Supplementary material for: Patterns of attention deficit in relapsing and progressive phenotypes of multiple sclerosis
Source: Sci Rep. 2023 Aug 10;13:13045. doi: 10.1038/s41598-023-40327-x (PMC10415341; doi:10.1038/s41598-023-40327-x)
Supplement: Supplementary file 1 — Supplementary Information. [file 41598_2023_40327_MOESM1_ESM.docx]

**Supplementary Table S1.**

*Current Disease Modifying Drugs of Multiple Sclerosis Patients.*

| Task | Phenotype | Disease modifying drug | Number (percent) |
| --- | --- | --- | --- |
| ANT | RRMS | Interferon Beta | 5 (16.66) |
|  |  | Dimethyl Fumarate | 2 (6.66) |
|  |  | Fingolimod | 1 (3.33) |
|  |  | Natalizumab | 1 (3.33) |
|  |  | Rituximab | 20 (66.66) |
|  |  | No Treatment | 1 (3.33) |
|  | SPMS | Ocrelizumab | 2 (6.25) |
|  |  | Rituximab | 30 (93.75) |
| PSCT | RRMS | Interferon Beta | 4 (20) |
|  |  | Dimethyl Fumarate | 1 (5) |
|  |  | Fingolimod | 1 (5) |
|  |  | Rituximab | 13 (65) |
|  |  | No Treatment | 1 (5) |
|  | SPMS | Ocrelizumab | 1 (4.76) |
|  |  | Rituximab | 20 (95.24) |

ANT: attention network test, PSCT: Posner spatial cueing test, RRMS: relapse-remitting multiple sclerosis, SPMS: secondary progressive multiple sclerosis.

**Supplementary Table S2.**

*Significant Differences Between Results of Participants in Attention Network Test.*

| Reaction time or error rate | SPMS | RRMS | HC | p-value* | MS | p-value** |
| --- | --- | --- | --- | --- | --- | --- |
| No Cue Congruent RT | 731.96 ± 148.74 | 649.17 ± 86.35 | 614.18 ± 118.74 | (SPMS vs. HC) 0.001 | 691.90 ± 130.43 | 0.004 |
| Center Cue Congruent RT | 686.30 ± 162.77 | 606.19 ± 90.72 | 573.19 ± 118.82 | (SPMS vs. HC) 0.004 | 647.54 ± 139.91 | 0.007 |
| Double Cue Congruent RT | 665.12 ± 153.12 | 601.75 ± 85.50 | 552.82 ± 104.94 | (SPMS vs. HC) 0.002 | 634.46 ± 130.06 | 0.001 |
| Spatial Cue Congruent RT | 657.75 ± 154.70 | 603.97 ± 80.42 | 554.37 ± 108.65 | (SPMS vs. HC) 0.007 | 631.73 ± 128.33 | 0.003 |
| No Cue Incongruent RT | 828.91 ± 159.21 | 765.25 ± 111.18 | 725.79 ± 121.29 | (SPMS vs. HC) 0.014 | 798.11 ± 142.84 | 0.013 |
| Center Cue Incongruent RT | 826.77 ± 172.21 | 760.55 ± 97.72 | 718.81 ± 126.20 | (SPMS vs. HC) 0.018 | 794.73 ± 146.18 | 0.012 |
| Double Cue Incongruent RT | 786.02 ± 175.78 | 722.16 ± 96.65 | 677.16 ± 124.04 | (SPMS vs. HC) 0.012 | 755.12 ± 147.78 | 0.007 |
| No Cue Neutral RT | 692.60 ± 134.91 | 638.06 ± 83.24 | 599.44 ± 108.72 | (SPMS vs. HC) 0.007 | 666.21 ± 117. 09 | 0.007 |
| Center Cue Neutral RT | 662.00 ± 151.78 | 593.44 ± 93.28 | 555.59 ± 102.63 | (SPMS vs. HC) 0.002 | 628.83 ± 132.50 | 0.004 |
| Double Cue Neutral RT | 641.84 ± 151.32 | 582.56 ± 82.88 | 554.66 ± 95.84 | (SPMS vs. HC) 0.022 | 613.15 ± 127.60 | 0.016 |
| Spatial Cue Neutral RT | 644.87 ± 146.57 | 580.42 ± 83.47 | 542.11 ± 104.50 | (SPMS vs. HC) 0.006 | 613.68 ± 125.50 | 0.008 |
| Double Cue Neutral ER | 0.95 ± 2.16 | 0.14 ± 0.75 | 0 | (SPMS vs. HC) 0.014 | 0.56 ± 1.7 | 0.066 |
| Total RT | 716.53 ± 154.52 | 649.38 ± 82.84 | 612.93 ± 109.01 | (SPMS vs. HC) 0.006 | 684.04 ± 130.56 | 0.007 |

All data are presented as mean ± standard deviation. Reaction time (RT) and error rate (ER) are shown in millisecond and percent, respectively, and classified by the type of cue (no cue, center, double, and spatial) and target (neutral, congruent, and incongruent). Significance level is 0.05, using Kruskal Wallis test and Tukey Kramer post hoc analysis. *: this column shows the p-value of comparison between HC, RRMS, and SPMS; groups with significant differences are written in parenthesis. **: this column shows the p-value of comparison between HC and all MS patients (RRMS and SPMS). SPMS: secondary progressive MS, RRMS: relapse-remitting MS, MS: multiple sclerosis, HC: healthy control.

**Supplementary Table S3.**

*Attentional Network Effects of Participants in Attention Network Test.*

| Attentional network effect | SPMS | RRMS | HC | p-value* | MS | p-value** |
| --- | --- | --- | --- | --- | --- | --- |
| Differential Alerting Effect | 53.49 ± 26.92 | 48.67 ± 22.61 | 51.10 ± 16 | 0.261 | 51.16 ± 25.25 | 0.841 |
| Differential Orienting Effect | 32.72 ± 35.88 | 28.90 ± 35.76 | 28.91 ± 21.27 | 0.717 | 30.87 ± 36.17 | 0.734 |
| Differential Executive Effect | 118.71 ± 49.30 | 119 ± 56.35 | 111.03 ± 29.91 | 0.978 | 118.85 ± 53.26 | 0.955 |
| Proportional Alerting Effect | 0.08 ± 0.04 | 0.07 ± 0.03 | 0.08 ± 0.03 | 0.418 | 0.08 ± 0.04 | 0.48 |
| Proportional Orienting Effect | 0.049 ± 0.05 | 0.044 ± 0.05 | 0.05 ± 0.03 | 0.956 | 0.05 ± 0.05 | 0.993 |
| Proportional Executive Effect | 0.17 ± 0.06 | 0.18 ± 0.08 | 0.19 ± 0.05 | 0.185 | 0.17 ± 0.07 | 0.131 |
| Residual Alerting Effect | 52.64 ± 26.75 | 48.68 ± 22.67 | 52.72 ± 22.02 | 0.371 | 50.72 ± 25.14 | 0.894 |
| Residual Orienting Effect | 31.87 ± 35.85 | 28.91 ± 35.8 | 26.31 ± 22.95 | 0.652 | 30.43 ± 36.15 | 0.527 |
| Residual Executive Effect | 117.84 ± 49.41 | 118.98 ± 56.35 | 128.07 ± 46.69 | 0.463 | 118.39 ± 53.31 | 0.224 |

All data are presented as mean ± standard deviation. *: significance level is 0.05, using Kruskal Wallis test. *: this column shows the p-value of comparison between HC, RRMS, and SPMS groups. **: this column shows the p-value of comparison between HC and all MS patients (RRMS and SPMS). SPMS: secondary progressive MS, RRMS: relapse-remitting MS, MS: multiple sclerosis, HC: healthy control.

**Supplementary Figure S1.**

*Reaction Time of Participants in Posner Spatial Cueing Test.*


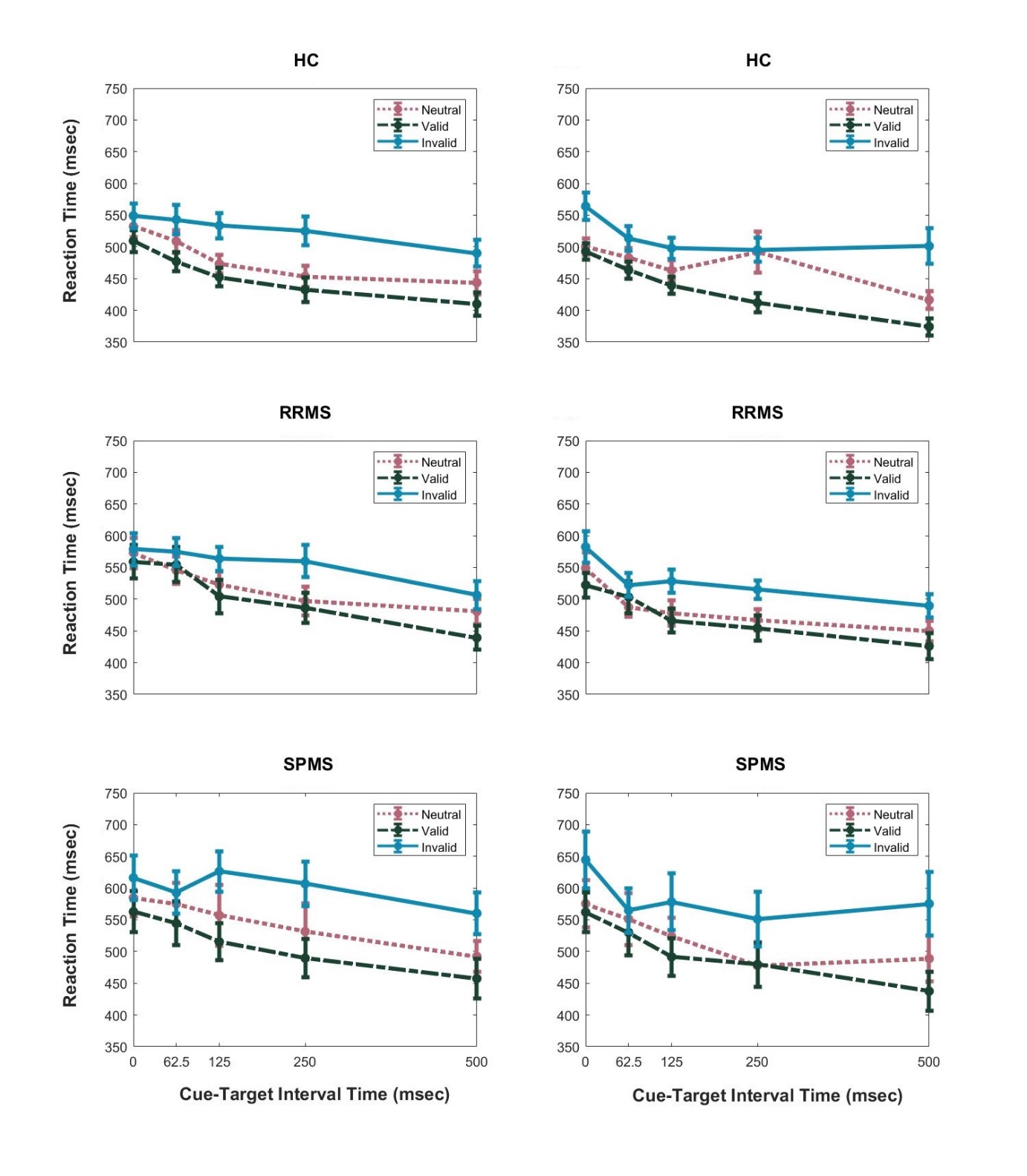


Reaction time is presented in milliseconds. Error bars are between subject ± 1 standard error of mean. Results in each group (top: HC, middle: RRMS, and bottom: SPMS) were classified by the type of block (exogenous on left and endogenous on right), cue (neutral, valid, and invalid), and cue-target interval time (0, 62.5, 125, 250, and 500 msec). No statistical difference was observed between groups. HC: healthy control, RRMS: relapse-remitting multiple sclerosis, SPMS: secondary progressive multiple sclerosis, msec: millisecond.

**Supplementary Figure S2.**

*Error Rate of Participants in Posner Spatial Cueing Test.*


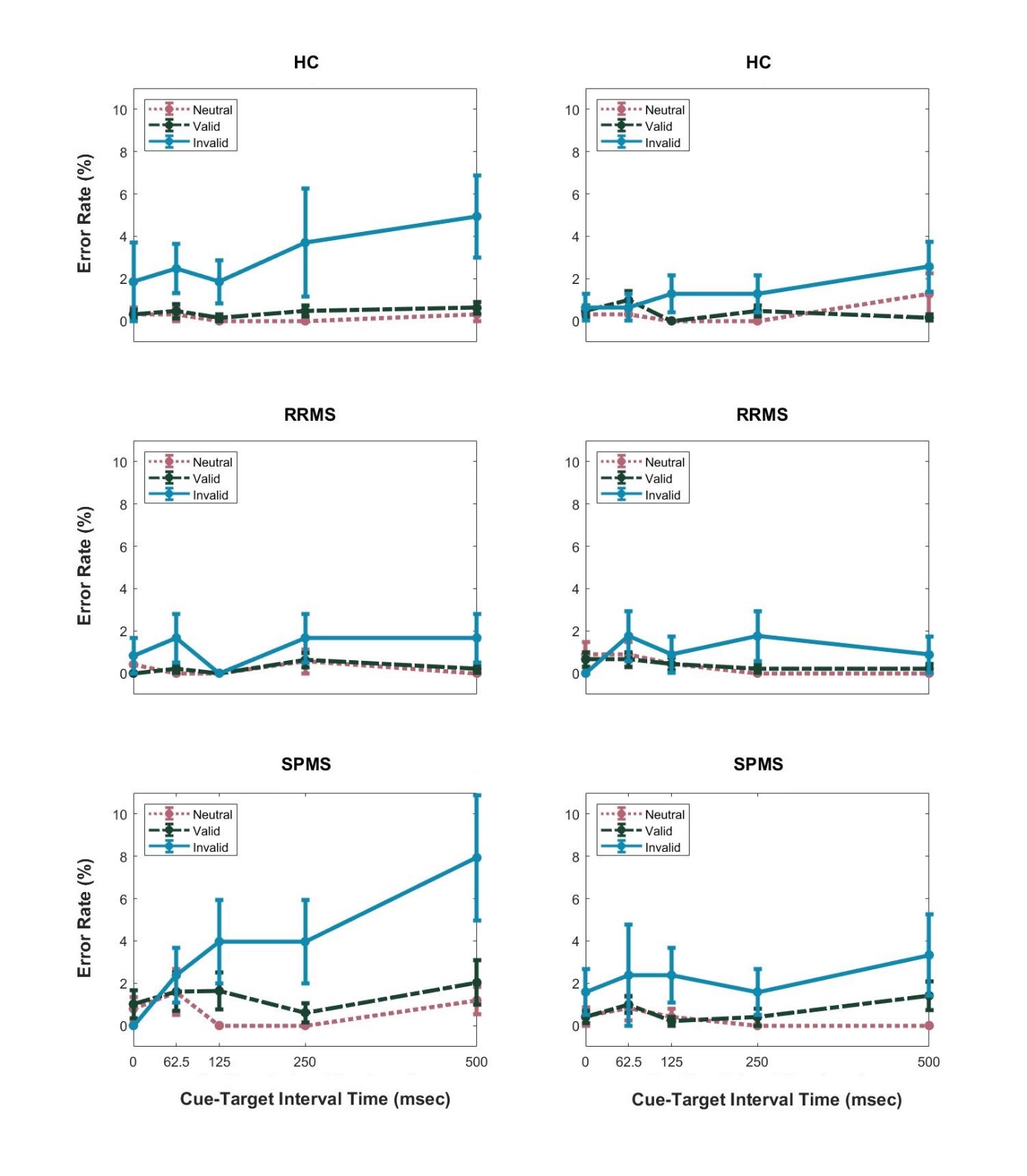


Error rate is presented in percent. Error bars are between subject ± 1 standard error of mean. Results in each group (HC, RRMS, and SPMS) are classified by the type of block (exogenous on left and endogenous on right), cue (neutral, valid, and invalid), and cue-target interval time (0, 62.5, 125, 250, and 500 msec). Error rate of 125 and 500 msec cue-target interval time were significantly higher in SPMS than RRMS patients. HC: healthy control, RRMS: relapse-remitting multiple sclerosis, SPMS: secondary progressive multiple sclerosis, msec: millisecond.

**Supplementary Table S4.**

*Gain and Cost of Participants in Posner Spatial Cueing Test.*

| Gain or cost | SPMS | RRMS | HC | p-value* | MS | p-value** |
| --- | --- | --- | --- | --- | --- | --- |
| Exogenous Total Gain | -34.27 ± 42.96 | -15.24 ± 36.90 | -26.01 ± 33.45 | 0.452 | -24.99 ± 41.74 | 0.856 |
| Exogenous Gain in 0ms CTIT | -21.98 ± 81.46 | -14.19 ± 58.17 | -22.96 ± 48.51 | 0.944 | -18.18 ± 72.05 | 0.759 |
| Exogenous Gain in 62.5ms CTIT | -30.85 ± 62.10 | 8.86 ± 82.58 | -31.57 ± 53.43 | (RRMS vs. HC)  0.048 * | -11.48 ± 76.41 | 0.107 |
| Exogenous Gain in 125ms CTIT | -41.91 ± 110.30 | -18.66 ± 84.35 | -21.06 ± 39.89 | 0.796 | -30.57 ± 100.41 | 0.568 |
| Exogenous Gain in 250ms CTIT | -41.79 ± 101.07 | -10.61 ± 57.16 | -20.89 ± 58.43 | 0.705 | -26.58 ± 85.12 | 0.905 |
| Exogenous Gain in 500ms CTIT | -34.82 ± 74.35 | -41.61 ± 38.86 | -33.59 ± 58.20 | 0.614 | -38.13 ± 60.57 | 0.502 |
| Exogenous Total Cost | 52.26 ± 58.37 | 32.78 ± 36.96 | 45.85 ± 37.02 | 0.147 | 42.76 ± 50.68 | 0.817 |
| Exogenous Cost in 0ms CTIT | 31.402 ± 127.15 | 5.62 ± 75.50 | 16.73 ± 48.52 | 0.541 | 18.82 ± 107.28 | 0.620 |
| Exogenous Cost in 62.5ms CTIT | 17.48 ± 92.60 | 29.04 ± 94.63 | 34.24 ± 90.76 | 0.934 | 23.12 ± 94.94 | 0.730 |
| Exogenous Cost in 125ms CTIT | 69.23 ± 152.93 | 40.85 ± 89.48 | 60.37 ± 70.28 | 0.213 | 55.39 ± 128.41 | 0.721 |
| Exogenous Cost in 250ms CTIT | 75.31 ± 97.13 | 62.84 ± 82.78 | 71.42 ± 89.04 | 0.525 | 69.22 ± 91.76 | 0.552 |
| Exogenous Cost in 500ms CTIT | 67.87 ± 76.20 | 25.55 ± 69.02 | 46.49 ± 62.60 | 0.127 | 47.23 ± 76.74 | 0.856 |
| Endogenous Total Gain | -23.28 ± 28.07 | -11.91 ± 26.53 | -34.36 ± 40.51 | 0.192 | -17.88 ± 28.29 | 0.081 |
| Endogenous Gain in 0ms CTIT | -13.30 ± 62.74 | -26.62 ± 64.97 | -7.69 ± 38.09 | 0.453 | -19.63 ± 64.97 | 0.462 |
| Endogenous Gain in 62.5ms CTIT | -21.21 ± 48.48 | 15.94 ± 61.22 | -19.55 ± 58.08 | 0.159 | -3.56 ± 58.69 | 0.338 |
| Endogenous Gain in 125ms CTIT | -32.21 ± 46.90 | -11.64 ± 31.90 | -23.76 ± 45.62 | 0.473 | -22.44 ± 42.29 | 0.896 |
| Endogenous Gain in 250ms CTIT | 1.66 ± 65.55 | -13.00 ± 72.93 | -79.24 ± 156.32 | (SPMS vs. HC)  0.032 * | -5.30 ± 70.42 | 0.016 ** |
| Endogenous Gain in 500ms CTIT | -51.36 ± 49.36 | -24.25 ± 42.38 | -42.30 ± 37.36 | 0.132 | -38.48 ± 48.74 | 0.646 |
| Endogenous Total Cost | 59.41 ± 43.78 | 41.18 ± 40.95 | 43.79 ± 50.77 | 0.315 | 50.75 ± 43.98 | 0.637 |
| Endogenous Cost in 0ms CTIT | 69.15 ± 104.68 | 33.43 ± 100.79 | 63.48 ± 61.62 | 0.448 | 52.18 ± 105.72 | 0.763 |
| Endogenous Cost in 62.5ms CTIT | 13.67 ± 62.22 | 34.97 ± 61.04 | 30.91 ± 77.18 | 0.544 | 23.79 ± 63.37 | 0.753 |
| Endogenous Cost in 125ms CTIT | 54.52 ± 124.74 | 50.37 ± 61.64 | 35.01 ± 71.64 | 0.460 | 52.55 ± 101.16 | 0.600 |
| Endogenous Cost in 250ms CTIT | 73.28 ± 81.68 | 47.73 ± 70.74 | 3.58 ± 159.01 | 0.079 | 61.15 ± 78.72 | 0.033 ** |
| Endogenous Cost in 500ms CTIT | 86.43 ± 87.32 | 39.39 ± 48.83 | 84.88 ± 121.96 | 0.123 | 64.08 ± 76.38 | 0.462 |

All data are presented in milliseconds as mean ± standard deviation classified by the type of block (exogenous, endogenous) and CTIT (0, 62.5, 125, 250, and 500 ms). Significance level is 0.05, using Kruskal Wallis test and Tukey Kramer post hoc analysis. *: this column shows the p-value of comparison between HC, RRMS, and SPMS; significant p-values are marked by * and groups with significant differences are written in parenthesis. **: this column shows the p-value of comparison between HC and all MS patients (RRMS and SPMS); significant p-values are marked by **. SPMS: secondary progressive MS, RRMS: relapse-remitting MS, HC: healthy control, MS: multiple sclerosis, ms: millisecond, CTIT: cue-target interval time.

**Supplementary Figure S3.**

*Capacity of Gain in Posner Spatial Cueing Test for Discrimination Between Multiple Sclerosis Phenotypes.*

**
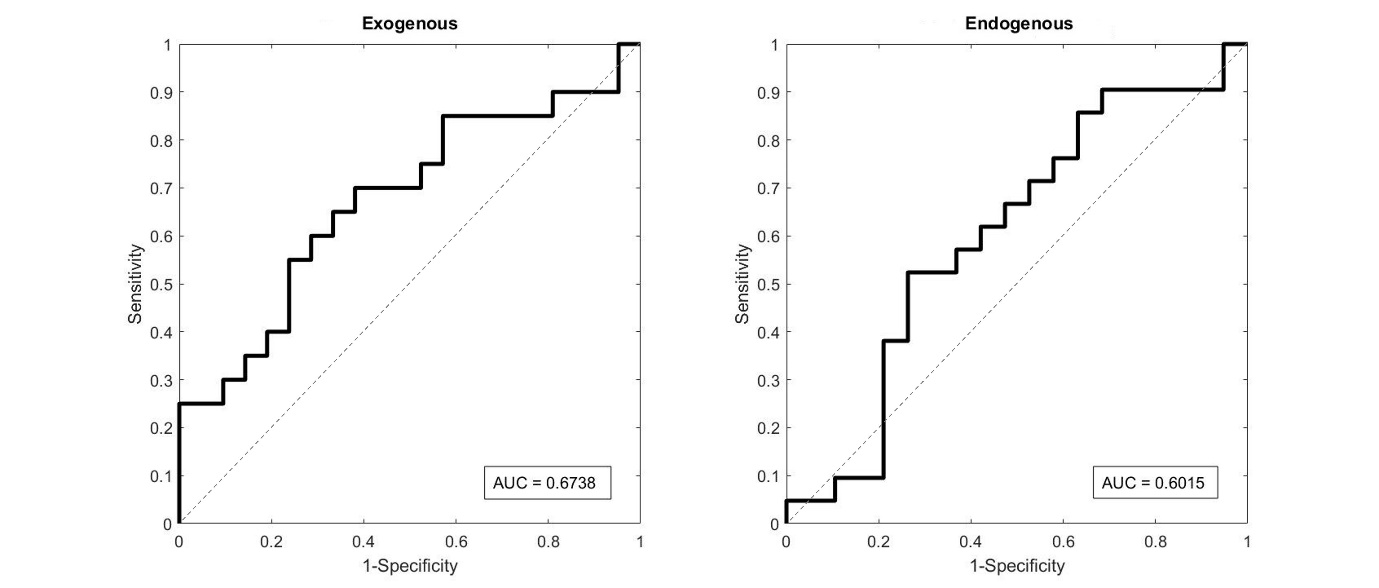
**

Receiver operating curve is plotted for discriminating relapse-remitting multiple sclerosis (RRMS) from secondary progressive multiple sclerosis (SPMS) by gain in 62.5ms in the exogenous Posner task (left) or gain in 250ms in the endogenous Posner task (right). Area under curve (AUC) is written on each subplot.
